# LFF-POS: A linguistic fusion method to handle out-of-vocabulary words in low-resource part-of-speech tagging

**DOI:** 10.1016/j.mex.2025.103615

**Published:** 2025-09-10

**Authors:** Muhammad Alfian, Umi Laili Yuhana, Daniel Siahaan, Harum Munazharoh, Eric Pardede

**Affiliations:** a Department of Informatics, Institut Teknologi Sepuluh Nopember, Surabaya 60112, Indonesia; b Department of Indonesian Language and Literature, Universitas Airlangga, Surabaya 60286, Indonesia; c Department of Computer Science and Information Technology, La Trobe University, Melbourne 3086, Australia

**Keywords:** Out-of-vocabulary, Deep learning, Part-of-speech tagging, Low-resource language, Quality of education, Morphological-rich language

## Abstract

Accurate part-of-speech (POS) tagging is needed for classroom learning evaluation in order to improve the quality of education. However, accurate POS tagging is hampered by the limited amount of training data and the high proportion of out-of-vocabulary (OOV) tokens. We present LFF-POS, a linguistic feature fusion method that overcomes these limitations for Indonesian. The procedure consists of four sequential steps: (1) tokenizing raw text; (2) extracting three complementary features; (3) merging the resulting vectors; (4) applying self-attention; and (4) training a BiLSTM sequence labeler. By combining the three features, LFF-POS improves tagging accuracy without relying on an external lexicon. Experimental results show that the combined features are able to improve the proposed model's ability to handle OOV words and achieve higher POS Tagging accuracy compared to baseline and existing methods.

OOV cannot be recognized by the model, thus reducing the accuracy of the POS Tagging model

This study aims to overcome OOV by combining linguistic features such as orthography, morphology, and characters to improve word representation

The LFF-POS has been proven to improve POS Tagging performance, especially OOV F1 Score by ±14% over baseline.

## Graphical abstract

**Figure:**
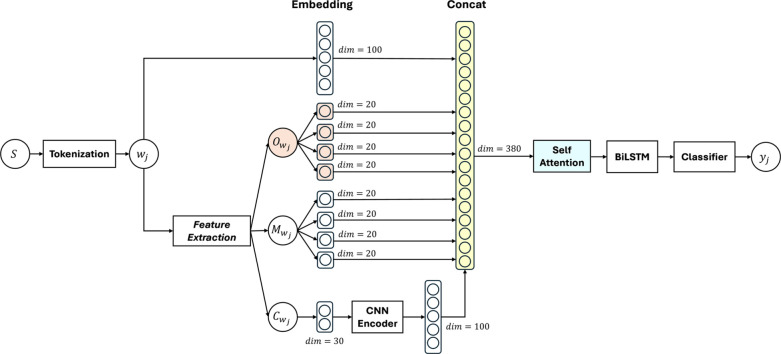

**Specifications table**

**Table:** 

**Subject area**	Computer Science
**More specific subject area**	Computational Linguistic, Natural Language Processing, Deep Learning
**Name of your method**	LFF-POS: A Linguistic Feature Fusion method to handle out-of-vocabulary words in low-resource Part-of-Speech tagging
**Name and reference of original method**	K. Kurniawan and A. F. Aji, “Toward a standardized and more accurate Indonesian Part-of-Speech tagging,” Proceedings of the 2018 International Conference on Asian Language Processing, IALP 2018, pp. 303–307, 2019, doi: 10.1109/IALP.2018.8629236.
**Resource availability**	• https://pytorch.org/ • https://pypi.org/project/torchtext/ • https://github.com/ir-nlp-csui/aksara • https://github.com/kmkurn/id-pos-tagging

## Background

POS Tagging is a fundamental task in Natural Language Processing (NLP) [1]. POS is a grammatical classification that typically includes several word classes, such as verbs, adjectives, adverbs, and nouns [2]. It is used for various downstream NLP tasks, such as sentence parsing [3], text classification [4], and text summarization [5]. The word class information generated by POS Tagging has been shown to improve accuracy on other NLP tasks [4]. However, the latest POS Tagging model is still not optimal due to the out-of-vocabulary (OOV) issue [6]. OOV are terms that do not exist in the training vocabulary but appear in the testing phase, so the model cannot predict the label accurately. Handling OOV can directly improve the accuracy of the POS Tagging model and indirectly help other researchers in improving the performance of other NLP tasks such as sentence parsing, text classification, and text summarization. For example, Leung et al. [7] used POS Tagging for examining the collocation and colligational patterns used by teachers and students during lessons. In addition, accurate POS Tagging is also useful for evaluating the level of linguistic competence [8] and providing feedback for grammar correction [9]. So increasing the accuracy of POS Tagging can indirectly help improve the quality of education.

The OOV words often occur when analyzing conversational text, such as dialogs or tweets [10], in which unfamiliar terms are frequently encountered [11]. OOV usually occurs in the case of low-resource languages such as Ainu [12], Uyghur [13], and Indonesian [14]. The limited amount of annotated data in the low-resource language limits the model’s vocabulary coverage. OOV also occurs in morphologically rich languages such as Indonesian [15], Turkish [16], and Uzbek [6]. These languages have unique morphological information; thus, it is difficult to handle OOV in multiple languages simultaneously. These challenges highlight the importance of understanding how different linguistic and contextual factors contribute to the OOV phenomenon in POS tagging.

The latest study on low resource POS tagging in Indonesian language proposed a combination of morphological and character features with the bidirectional long short-term memory (BiLSTM) model to improve model accuracy [17]. However, this feature is still not enough to handle OOV words because not all OOV words have affixes, such as name entities [18]. Entity names such as people's names, geographical names, and other entity names have special word forms and cannot be recognized only by affixes and character arrangements. Therefore, studies related to OOV word representation are still open for further research. This study explores alternative features that can improve OOV word representation.

## Method details

**Fig. 1:**
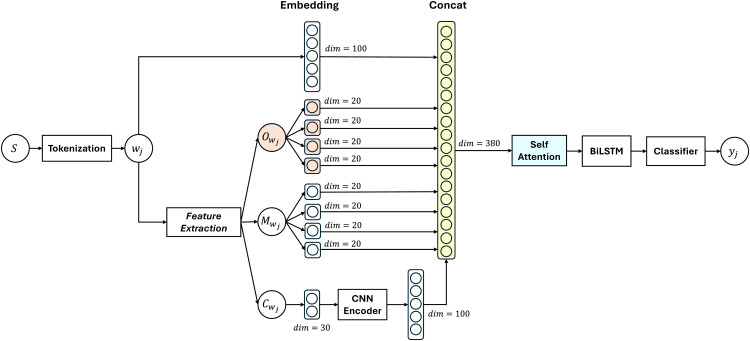
Architecture of the proposed model using the linguistic feature fusion and BiLSTM for enhanced OOV handling in Low-Resource POS tagging.

This study proposes a linguistic feature fusion that combines orthographic, morphological, and character features to improve the performance of the POS tagging model as shown in Fig 1. We adopt the architecture from Kurniawan and Aji's research [17] which uses morphological and character features to represent words. We modify the morphological features and add orthographic features that can improve the representation of OOV words. We extracted orthographic features such as capital letters, symbols, and numbers, by using modified technique proposed by Manning [19]. The objective is to recognize the characteristics of name entities which usually consist of special forms such as capital letters, numbers, or symbols. Morphological features were extracted using two different methods and were tested to determine which method produced higher POS tagging performance. The first method extracts affixes (prefix and suffix) in each word. The second method extracts affixes using formal morphological analyzer tools, Aksara. Formal morphological feature extraction is expected to improve word representation. Character features were extracted from character-level embedding by using convolutional layers following the work of Kurniawan and Aji [17]. Several studies in other languages have demonstrated that character features can improve POS tagging performance [20]. All these linguistic features were combined using the concat method so that the total dimension of one word is 380 as highlighted in Fig 1. We also modified the model by adding a self-attention layer before the BiLSTM layer. The Self-Attention layer helps the model recognizing global context information in a sentence and enriches word information before being processed by BiLSTM. The BiLSTM model has been proven optimal for various cases of sequential labeling, including POS tagging [2]. We also compared two types of classification layers: softmax and conditional random fields (CRF). This study used a larger corpus than in previous similar studies, with a total of 355,021 words and a vocabulary of 29,031 words [21].

In the following, we detail our proposed method to improve Indonesian POS tagging performance. Fig. 1 presents the overall study stages. The stages are as follows: 1) breaking the input sentence (*S_i_*) into a list of words (*W*) (tokenization), 2) extracting features based on orthography, morphology, and characters, 3) and combining the features using concatenation, 4) recognizing global context with self-attention, 5) recognizing local context and word order patterns with BiLSTM, 6) obtaining word classes with classifier, 7) evaluating the POS tagging model and assessing its ability in OOV handling. These stages are explained in greater detail in the following subsections.

### Dataset

The dataset used in this study was adopted from a previous study on Indonesian POS tagging [21], which consists of 21,024 sentences with 355,021 words. The corpus uses a tagset comprising of 29 tags. We grouped the tagset into word categories and word classes contained in Standard Indonesian Grammar as shown in Table 1.

**Table 1:** List of word classes in the corpus [21].

Category	Label	Word Class	Word List
Noun	NN	Common Noun	*buku, pipi, rupiah, km, sekarang*
	NNP	Proper Noun	*Indonesia, MH370, Li, SBY*
	SP	Subject-predicate structure	*katanya, sebutnya, tuturnya*
Pronoun	PRD	Demonstrative Pronoun	*ini, itu, sini, sana, tersebut*
	PRF	Reflexive Pronoun	*sendiri, diri, dirinya*
	PRI	Indefinite Pronoun	*siapapun, apapun, seseorang*
	PRL	Relative Pronoun	*yang*
	PRP	Personal Pronoun	*saya, kamu, dia, kami, kalian*
	WH	Question	*apa, siapa, mana, bagaimana*
Adjective	JJ	Adjective	*besar, tinggi, manis, cerdik*
	JJS	Adjective, superlative	*terdekat, terbesar, terpenting*
Verbs	VB	Verb	*ada, melihat, gagal, menyoroti*
	VO	Verb-object structure	*meningkatnya, terbentuknya*
Adverbs	MD	Auxilary Verb	*harus, perlu, boleh, adalah, mau*
	RB	Adverb	*sudah, tidak, sangat, juga*
Conjunction	CC	Coordinating Conjunction	*dan, tetapi, atau*
	SC	Subordinating Conjunction	*kalau, jika, sementara itu*
Interjection	IN	Preposition	*di, ke, oleh, untuk, dari, antara*
	PO	Preposition Object Structure	*untuknya, antaranya, olehku*
Determiner	UH	Interjection	*oh, hai, ya, sih, mari*
	DT	Determiner	*para, sang, si*
	CD	Cardinal Number	*satu, dua, 79, 2017, 0.1, ratus*
	OD	Ordinal Number	*pertama, ketiga, ke-6*
	ID	Indefinite Number	*puluhan, 30-an, beberapa*
Particle	P	Particle	*pun, -lah, -kah*
Symbols	SYM	Symbol	*+,%, @, $, 15/2/2017, 13:00, Rp*
	Z	Punctuation	*“,.?”()*
Miscellaneous	FW	Foreign Word	*poetry, technology, out, world*
	X	Unknown	*yagg, busaway, saaat*

We used cross-validation to split the dataset into three sets: training (train), testing (test), and validation/development (dev). The folds were set at five and the training-to-testing data ratio was 80:20. Table 2 shows the distribution of sentences, words, vocabulary, and OOV words in each fold. We randomly selected 10 % of the sentences in the training set to be used as the validation (dev). Therefore, OOV words were calculated from the number of words that were not encountered in the training (train).

**Table 2:** Data Distribution.

Fold	Dataset	Sent	Word	Vocab	OOV
1	Train Dev	15k 1.6k	255k 28.2k	24.8k 7.2k	- 1.2k
	Test	4.2k	70.8k	12.7k	3.1k
2	Train Dev	15k 1.6k	255k 28.4k	24.7k 73k	- 1.3k
	Test	4.2k	72.0k	12.7k	3.1k
3	Train Dev	15k 1.6k	256k 28.0k	24.7k 7.2k	- 1.2k
	Test	4.2k	70.7k	12.5k	3.1k
4	Train Dev	15k 1.6k	255k 28.0k	24.7k 7.2k	- 1.2k
	Test	4.2k	71.6k	12.7k	3.1k
5	Train Dev	15k 1.6k	257k 27.9k	24.9k 7.3k	- 1.3k
	Test	4.2k	69.7k	12.3k	2.9k

As shown in Table 2, the number of vocabulary words and OOV words was similar in each fold, except for the fifth fold. The number of OOV words in the testing set of the fifth set was approximately 2900, whereas the other folds contained approximately 3100 OOV words. This is also the case for the total number of words and the number of words within the vocabulary in the testing set, of which are lower in the fifth fold compared to the other four folds. This difference is due to the cross-validation technique, which divides the data based on the given sentences. The lower number of OOV words and words within the vocabulary in the testing set of the fifth fold was because long sentences within the dataset were mostly found in the training set of the fifth fold. The ratio of OOV words to total words was 1:20.

### Feature extraction

Given an input sentence $S_{i}=\;w_{j}\;\ldots \;w_{n}$, where $w_{j}$ is the *j-*th word, we extract the orthographic ($O_{w_{j}}$), morphological ($M_{w_{j}}$), and character ($C_{w_{j}}$) features.

Our proposed orthographic features adopt the features used by Manning [19] to build English POS Tagging. We adopt the features of word shape, capital letters, symbols, and numbers which are respectively symbolized as $a_{j}=encode\left(w_{j}\right)$, $b_{j}=isupper\left(w_{j}\right)$, $c_{j}=isnumber\left(w_{j}\right)$, and $d_{j}=issymbol\left(w_{j}\right)$. The wordshape feature is obtained from the coding process illustrated in Algorithm 1. Finally, the orthographic feature of each word is derived as shown in Eq. (1):

(1) $$O_{w_{j}}=\;\left\{a_{j},b_{j},\;c_{j},\;d_{j}\right\}$$

**Algorithm 1:** Word shape Encoding (a).

1	**Input:** the j-th word in sentences ($w_{j})$
2	**Output:** the j-th word shape in sentences ($a_{j}$)
3	**Initialization:** array of word shape in j-th word **(**$a_{jk}$)
4	**for**k=1**to**$length\;(w_{j})$**do** {iterations}
5	**if**$w_{jk}\;is\;numeric$**then**$a_{jk}$*=“d”*
6	**else if**$w_{jk}$*is not alphanumerics***then**$a_{jk}$*=*$w_{jk}$
7	**else if**$w_{jk}$*is uppercase***then**$a_{jk}$*=“X”*
8	**else if**$w_{jk}$*is lowercase***then**$a_{jk}$*=“x”*
9	**end if**
12	**end for**

Morphological features were extracted using two approaches. The first approach intuitively extracts prefixes ($p_{n}$) dan suffixes ($s_{n}$) with an n-gram approach. We use two variations of subwords with n=2 dan n=3. This technique assumes that every word has an affix. The morphological features extracted using this approach are derived as shown in Eq. (2):

(2) $$M_{w_{j}}=\left\{p_{2},\;p_{3},\;s_{2},\;s_{3}\right\}\;$$

The second method extracts morphological features using the Aksara tool, which is based on linguistic theory and conforms to standard Indonesian grammar [22]. This approach converts the results of the tool [23] in CoNLL-U format, denoted by $e_{j}=Aksara\left(w_{j}\right)$, into vector/embedding form. Not all the script results were used in this study. The only variables related to morphosyntax used in this study are prefixes ($e_{j}^{p}$), lemmas ($e_{j}^{l}$), suffixes ($e_{j}^{s}$), and clitics ($e_{j}^{c}$). Occasionally, words with more than one prefix are found, thereby extending the vector. The resulting vector is derived as shown in Eq. (3):

(3) $$M{{}^{\prime }}_{w_{j}}=\left\{e_{j}^{p},\;e_{j}^{l},e_{j}^{s},e_{j}^{c}\right\}$$

Orthographic and morphological features are represented using embedding layers provided by the Pytorch library. Embedding layers automatically convert index values into vectors with dense layers. We also configure a dropout of 0.5 to avoid overfitting. The values of each vector are generated randomly with low dimensions (dim=20).

**Fig. 2:**
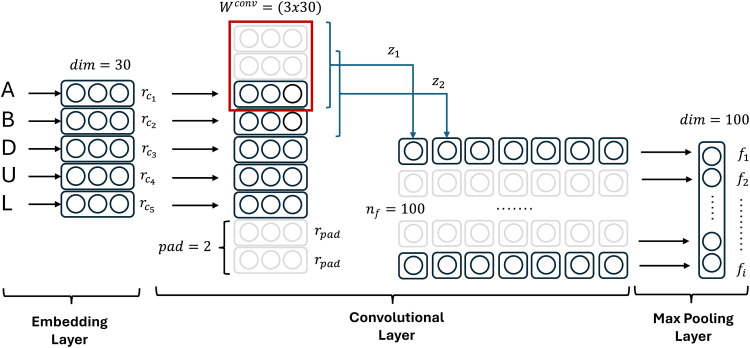
Architecture of CNN Encoder.

The character features were extracted from a word ($w_{j}$), consists of M characters $\left\{c_{1},\;c_{2},\;\ldots ,\;c_{M}\right\}$. Similar to orthographic and morphological features, each character is represented using an embedding layer with dim=30. As shown in Fig. 2, each character ($c_{m}$) is represented by character embedding ($r_{c_{m}}$). The character embedding is a vector of size $n_{c}$ with a value of 1 for index $c_{m}$ and 0 for other indices. Then, we added convolutional layers which the input is a sequence of character embeddings $\left\{r_{c_{1}},r_{c_{2}},\ldots ,r_{c_{M}}\right\}$. We also added two padding characters ($r_{pad}$) to anticipate the border effect. These two paddings are added at the beginning and end of each word $\left(w_{j}\right)$.

A convolutional filter ($W^{conv})$ applied over the sliding window $\left(r_{c_{m}-d_{c}+1}:\ldots :r_{c_{m}}\right)$ produces a local feature ($z_{a})$ that is derived as shown in Eq. (4):

(4) $$z_{a}=\;W^{conv}{\left(r_{c_{m}-d_{c}+1}:\ldots :r_{c_{m}}\right)}^{T}+b^{conv}$$

The length of the sliding window is determined by the $d_{c}$ parameter, which represents the number of characters. The local feature ($z_{a})$ is a vector of length $n_{f}$, which is obtained from each character ($c_{m})$ in each word ($w_{j})$. Local features ($z_{a})$ within a word ($w_{j})$ are combined using the max function as shown in Eq. (5):

(5) $${\left[f\right]}_{i}=\tanh\left(\max_{1<a<A}{\left[z_{a}\right]}_{i}\right)$$

Calculating the maximum function on each element (*i*) of the local feature ($x_{m}$) produces the character feature of the word $C=\{f_{1},f_{2},\ldots ,f_{n_{f}}\}$. The matrix $W^{conv}$ and vector $b^{conv}$ are the learned parameters. The character embedding length ($n_{c}$), feature length ($n_{f}$), and sliding window length ($d_{c}$) are the hyperparameters.

### Linguistic feature fusion

Finally, the fused feature ($x_{t}$) is obtained by combining the orthographic (O), morphological (M), and character (C) features, as shown in Eq. (6):

(6) $$x_{t}=\;O\;⊕\;M\;⊕\;C$$

It is important to note that morphological features use one of the two proposed approaches. Feature fusion cannot combine two types of the same morphological features. Therefore, during testing, morphological features are tested alternatively between intuitive morphological features (*M*) and morphological features using Aksara (*M*′).

### Self-Attention

**Fig. 3:**
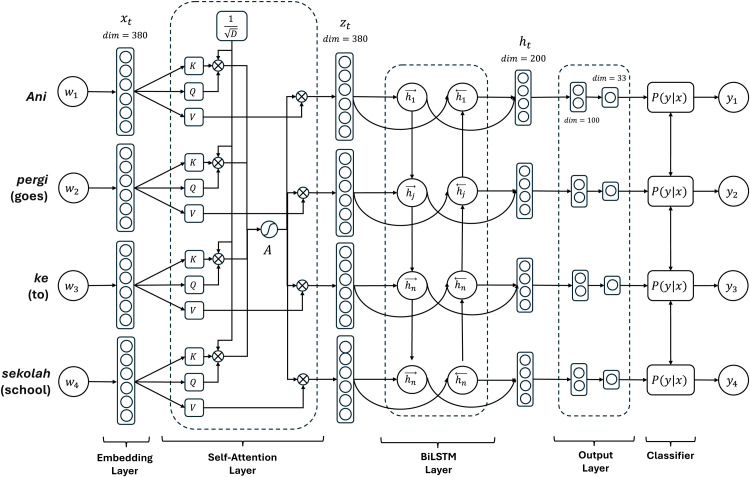
Architecture of Self-Attention, BiLSTM, and Classifier.

We use Self-Attention to capture the long-range dependencies of a word in a sentence. Fig. 3 shows the architecture of Self-Attention. It computes the weights ($A_{t}$) based on the relevance of $Q_{t}$ dan $K_{t}$. The Vectors $Q_{t}$, $K_{t}$, dan $V_{t}$ are obtained by processing the linguistic features ($x_{t}$) on the linear layer available in Pytorch and learning their weights (W) as shown in Eqs. (7)-(9).

(7) $$Q_{t}=x_{t}W^{Q}$$

(8) $$K_{t}=x_{t}W^{K}$$

(9) $$V_{t}=x_{t}W^{V}$$

$A_{t}$ is generated by processing the $Q_{t}$ and $K_{t}$ values ​​of all words in a sentence with a softmax layer as shown in Eq. (10). Meanwhile, the dimension (D) is the length of the linguistic features ($x_{t}$) with dim=380.

(10) $$A_{t}=softmax\left(\frac{Q_{t}{K_{t}}^{T}}{\sqrt{D}}\right)$$

The weights ($A_{t}$) emphasize the truly informative tokens and see which parts of the input are influential in the prediction. In this way, Self-Attention helps the model recognize OOV words by aligning the words with the surrounding context. The output of the Self-Attention layer is denoted by $z_{t}$ as shown in Eq. (11). The vector $z_{t}$ has the same dimension as its input (dim=380).

(11) $$z_{t}=A_{t}V_{t}$$

### BiLSTM

We exploit BiLSTM, a modification of the LSTM method, to generate high-level word representation. BiLSTM can process past and future information to capture contextual information. The hidden state ($h_{t}$) is the output of the BiLSTM model, expressed as $h_{t}=\;\vec{h_{t}}$⊕$\overset{\leftarrow }{h_{t}}$, where $\vec{h_{t}}$ is the sequence of past information, and $\overset{\leftarrow }{h_{t}}$ is the sequence of future information. The vector of the fused feature ($x_{t})$ is used as the input to the BiLSTM model, as shown in Eqs. (12)-(13).

(12) $$\vec{h_{t}}=\;\vec{LSTM}\left(z_{t},\;{\vec{h}}_{t-1}\right)$$

(13) $$\overset{\leftarrow }{h_{t}}=\;\overset{\leftarrow }{LSTM}\left(z_{t},\;{\overset{\leftarrow }{h}}_{t-1}\right)$$

The output of the BiLSTM layer is a vector $h_{t}$ (dim=200) as shown in Fig. 3. The vector $h_{t}$ is projected into the output layer which consists of two stages. In the first stage, the vector is projected into a vector (dim=100). This stage forces the model to extract the most informative features and prevents overfitting with additional dropout layers. In the second stage, the vector is projected into a vector (dim=33), corresponding to the POS Tagging labels with the model's default tags.

### Classifier

We employ a classification layer to decode the BiLSTM-generated values into word classes for POS tagging. We used two approaches in this study: a softmax layer with greedy decoding and the CRF layer. The softmax layer selects the word class by calculating the highest probability P(y) from the vector of the word class ($V^{y}$). The input to this layer is the output layer produced by BiLSTM, which is represented by $h_{t}$. The word class y is expressed as shown in Eq. (14):

(14) $$P\left(y=j|x\right)=\;\frac{e^{h_{j}}}{\sum_{k=0}^{K}e^{h_{k}}}$$

The CRF layer calculates the emission probability and models the transition probability for selecting an appropriate word class. Because the emission probability was calculated from the BiLSTM output layer, the CRF layer only learned the transition probability $A\in R^{K+2\;\times \;K+2}$, where k is the number of labels and 2 is the number of labels added at the beginning and end of the vector. The inference algorithm is derived as shown in Eq. (15):

(15) $$S\left(x,y\right)=\sum_{i=0}^{T}A_{y_{i},\;y_{i+1}}+\sum_{i=0}^{T}h_{i,y_{i}}$$

The probability of sequence y is represented as shown in Eq. (16):

(16) $$P\left(y|x\right)=\;\frac{e^{S\left(x,y\right)}}{\sum_{y^{\prime }\in y}e^{S\left(x,y^{\prime }\right)}}$$

The objective function is the maximum likelihood of the specified probability distribution, expressed as in Eq. (17):

(17) $$\ln P\left(y|x\right)=S\left(x,y\right)-\text{ln}\sum_{y^{\prime }\in y}e^{S\left(x,y^{\prime }\right)}$$

During training, the maximum likelihood of correct sequences is maximized. The final output tag sequence is determined based on the highest score, which is calculated as shown in Eq. (18):

(18) $$y^{*}\;=\;\underset{y^{\prime }\in y}{\text{argmax}}\;S\left(x,y^{\prime }\right)$$

### Evaluation models

The F1 score was used as the main evaluation method. The F1 score is shown in Eq. (19):

(19) $$F1=\;\frac{TP}{TP+\;\frac{1}{2}\;\left(FP+FN\right)}$$

We use two types of F1 score for evaluation: the sample-weighted F1 score (wF1) and the macro-averaged F1 score (aF1). Sample-weighted F1 score is used to assess model performance by considering the weight of each word class $w_{i}=n_{i}/N$, where $n_{i}$ is the number of samples in class i and N is the total sample. The sample-weighted F1 score is typically used to evaluate performance on imbalanced datasets. The formula for calculating the sample-weighted F1 score is shown in Eq. (20):

(20) $$wF1=\sum_{i=1}^{N}w_{i}\times F1_{i}$$

The macro-averaged F1 score evaluates performance by equally averaging the F1 scores of each word class. The macro-averaged F1 score is usually used to evaluate performance on balanced datasets. However, in this study, we use this metric to detect the slightest errors in each word class without considering its weight. This metric indicates the reliability of labeling all available word classes. The macro-averaged F1 score was calculated as shown in Eq. (21):

(21) $$aF1=\frac{\sum_{i=1}^{n}F1_{i}}{n}$$

We used both the sample-weighted F1 score and macro-averaged F1 score to comprehensively evaluate model performance. The sample-weighted F1 score fairly assesses model performance by weighting the contributions of each class according to their frequency in the dataset, thereby reflecting the impact of correct predictions on the overall data distribution. Meanwhile, the macro-averaged F1 score evaluates model performance across all classes equally, regardless of their frequency, highlighting errors in underrepresented or less frequent classes. This dual approach ensures a balanced assessment of the model's strengths and weaknesses.

## Method validation

In this section, we present and discuss in detail the experimental setup and results of the ablation study. The study examined the influence of the extracted features on the performance of the POS tagging model, specifically, how each feature contributed to handle OOV words.

### Ablation study of linguistic features

An ablation study was also conducted to investigate the influence of various features on the performance of the proposed POS tagging model. This experiment was run on a shared computer with an Intel i7–12,700 (2.10 GHz) processor, 96 GB of Random Access Memory (RAM), and an NVIDIA GeForce RTX 3080 Ti Graphics Card. The study was divided into four schemes: without the use of features (baseline model), with the use of one feature, with the use of a combination of two features, and with the use of a combination of three features.

The results are shown in Table 3. Based on the obtained OOV word classification results, the use of one feature resulted in higher wF1 scores compared to the baseline model. The wF1 score was calculated by considering the number of samples per class. Therefore, the wF1 score was highly dependent on the majority word class. Higher wF1 scores indicate that using one improved the ability of the model to classify the majority word class. However, using one feature resulted in lower aF1 scores than the baseline model. Both matrices obtained the same results for the softmax and CRF classification layers. The aF1 score calculation does not consider the number of samples per class. Thus, the low performance in classifying samples of the minority word class resulted in low aF1 scores. The lower aF1 scores indicate that the use of one feature is inadequate for handling OOV words, particularly OOV words of the minority word class.

**Table 3:** Result of Ablation Study.

Layer	Features				OOV		Overall	
	O	M	$M^{\prime }$	C	$wF_{1}$	$aF_{1}$	$wF_{1}$	$aF_{1}$
Softmax					69.70 %	37.65 %	92.97 %	81.08 %
	v				74.83 %	26.54 %	94.09 %	81.17 %
		v			81.54 %	33.91 %	94.67 %	83.78 %
			v		72.95 %	31.43 %	94.02 %	82.93 %
				v	81.75 %	35.80 %	94.68 %	84.68 %
	v	v			83.31 %	39.15 %	94.79 %	84.95 %
	v		v		80.40 %	37.11 %	94.58 %	84.35 %
	v			v	83.11 %	39.16 %	94.66 %	84.62 %
		v		v	83.63 %	40.10 %	94.84 %	85.21 %
			v	v	83.14 %	44.93 %	94.82 %	85.50 %
	v	v		v	84.05 %	42.26 %	94.82 %	85.24 %
	v		v	v	**84.06%**	**46.44%**	**94.93%**	**85.79%**
CRF					67.30 %	33.64 %	91.46 %	74.62 %
	v				74.57 %	26.46 %	94.05 %	80.75 %
		v			79.58 %	31.08 %	94.36 %	82.86 %
			v		72.21 %	29.02 %	93.96 %	82.90 %
				v	81.60 %	34.35 %	94.65 %	84.18 %
	v	v			83.63 %	40.46 %	94.81 %	84.94 %
	v		v		80.72 %	39.10 %	94.62 %	84.53 %
	v			v	82.99 %	38.91 %	94.71 %	85.07 %
		v		v	83.92 %	42.19 %	94.81 %	85.29 %
			v	v	83.19 %	42.09 %	94.86 %	85.37 %
	v	v		v	83.89 %	43.60 %	94.82 %	85.34 %
	v		v	v	**84.08%**	**45.05%**	**94.91%**	**85.53%**

Morphological and character features improved model performance when used as single features. The use of more than one feature resulted in higher wF1 and aF1 values compared to the baseline model, indicating the need to use more than one feature to achieve better performance. The use of these three features resulted in the best performance.

In addition, model performance was evaluated relative to classifying all words within the testing data and only OOV words in the testing data. In the classification of all words in the testing data, the three features significantly increased model performance. This is true for both softmax and CRF layers. The largest increase (by 10.91 % in performance was exhibited by the model that used CRF for classification, where the model achieved an aF1 score of 74.62 % without the use of features and achieved an aF1 score of 85.53 % with the use of three features. However, in the case of classifying OOV words within the testing data, the highest aF1 scores obtained by the model that used softmax and the model that used CRF were 46.44 % and 45.05 %, respectively. Although the use of three features increased the model performance by approximately 10 % compared to the baseline model, the obtained aF1 scores for both models were considered low.

The low *aF1* value is due to the model's inability to predict OOV words with minority labels, such as MD, CC, SC, DT, P, PO, and X. The model mispredicts minority labels because the features it has are not representative. This labeling error causes a decline in performance on downstream tasks, such as dependency parsing. Dependency parsing uses word class information to determine a single label for each input token of a sequence. Muñoz-Ortiz et al. [24] tested the effect of POS tagging performance on dependency parsing performance. The test results revealed a correlation between the performance of the POS tagging model and the quantity of the dataset on the performance of the dependency parsing model. This shows that the performance of the POS tagging model affects the performance of the model on other downstream tasks.

Next, we evaluated the tagging results between the model that does not use features (baseline) and our proposed model by observing sentences one by one. Table 4 presents an example of an observed sentence. The proposed model exhibits an improvement in tagging. The word “Davidson” is a named-entity that was initially recognized as a common noun (NN). After adding orthographic features, the model distinguished between common nouns (NN) and proper nouns (NNP). Then, the word “*Ditilang*” (ticketed) is a verb (VB). However, there was a typographical error in the sentence. The verb should be written in lowercase letters if it is in the middle of the sentence. The baseline model recognizes a word as a noun (NN) because it has a capital letter in front of it. However, due to the proposed morphological features, the proposed model can recognize the word as a verb (VB).

**Table 4:** Comparison of tagging results.

**Word**	*Pengguna*	*Harley*	*Davidson*	*Ditilang*	*di*	*HI*
	Rider of	Harley	Davidson	ticketed	at	HI
**Actual Label**	NN	NNP	NNP	VB	IN	NNP
**Prediction (Baseline)**	Z	NNP	NN	NN	IN	NNP
**Prediction (Proposed)**	NN	NNP	NNP	VB	IN	NNP
**Status**	**Improve**	Equal	**Improve**	**Improve**	Equal	Equal

### Classification layer comparison

In this study, the effect of the classification layer: softmax and CRF, on the results of classification was investigated. Table 3 shows the performance comparison between the POS tagging model that used CRF and the one that used softmax as the classification layer. It can be seen that without the use of features (baseline model), softmax slightly outperformed CRF by 6.46 %, with an aF1 score of 81.08 % (softmax) and 74.62 % (CRF). However, as more features are used, the performance gap decreased. For example, the model with one feature has the highest aF1 scores of 84.68 % (softmax) and 84.18 % (CRF) when using the character feature. The model with two features has the highest aF1 scores of 85.50 % (softmax) and 85.37 % (CRF) with the formal morphology and character features. The highest aF1 scores obtained using softmax and CRF were achieved using all three features. The model that used softmax achieved an aF1 score of 85.79 %, while the model that used CRF achieved an aF1 score of 85.53 % (CRF), indicating an insignificant performance gap of 0.26 %.

**Fig. 4:**
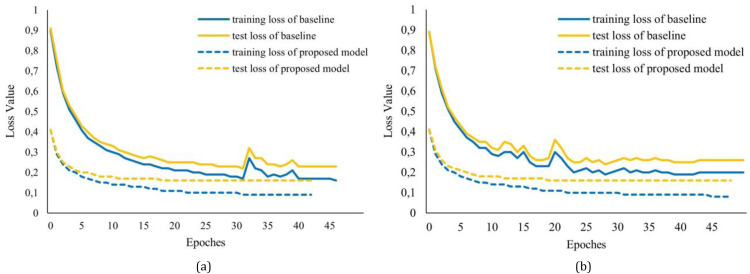
Training and test loss of (a) the baseline model and (b) the proposed model.

We further analyzed the performance of the proposed model by examining training and testing losses. Fig. 4.a shows the comparison of training loss and testing between the baseline model and proposed model that used softmax for classification. The training and testing loss curves show that the proposed model converged faster than the baseline model. This is also the case for the proposed model, which uses the CRF for classification (Fig. 4.b). It can be seen from Fig. 4.b that the proposed model, which uses the CRF, also converges faster than the baseline model. Furthermore, it can be seen in Fig. 4.a-b that the proposed model required a lower number of epochs than the baseline model. This indicates the high efficiency and stability of the proposed model, which leads to a faster learning process and decreases the number of training resources.

In addition, overfitting was analyzed by examining the training and test loss of each model. It can be seen in Fig. 4.a-b that the training loss continually decreased, whereas the testing loss did not decrease after 18 epochs for both the proposed model that used softmax and the proposed model that used CRF. Continuous training that results in a lower training loss but not testing loss can lead to model overfitting. Therefore, it is necessary to set parameters to determine when the model should stop training based on the difference between the training and testing losses.

### Previous method comparison

We compared the performance of the proposed method with several commonly used methods in POS Tagging, such as HMM, MEMM, CRF, and BiLSTM (baseline). We evaluated the performance of POS Tagging models in general and specifically in handling OOV words as shown in Table 5. The best POS Tagging model performance was achieved using the proposed method. The aF1 and wF1 values were superior to those of the previous methods. This proves the superiority of the proposed method in handling OOV words in Indonesian POS Tagging.

**Table 5:** Previous Model Evaluation.

Method	OOV		Overall	
	wF1	aF1	wF1	aF1
HMM	33.09 %	7.23 %	90.81 %	70.67 %
MEMM	75.36 %	21.63 %	89.30 %	63.60 %
CRF	66.93 %	30.69 %	90.42 %	75.90 %
BiLSTM (baseline)	69.70 %	37.65 %	92.97 %	81.08 %
BiLSTM (proposed)	84.06 %	46.44 %	94.93 %	85.79 %

## Limitations

In this paper, we proposed LFF-POS, a linguistic feature fusion method that extracts and combines orthographic, morphological, and character features to enhance Indonesian POS tagging. Using this feature fusion method, we construct a POS tagging model. Furthermore, two classification layers: softmax and CRF, were analyzed to determine the effect of the classification layer on model performance. We conducted an ablation study to investigate the effect of each single feature and combined features on model performance using an Indonesian POS tagging corpus that comprising 355,021 words. However, this study has limitations where the model still cannot label words correctly. We found unavoidable mislabeling of words with incorrect actual labels. In the future, we plan to develop models that use contextual information from pre-trained embeddings. This approach could help the recognition of words not found in the dictionary and overcomes labelling errors caused by a lack of representative features. Alfian et al. [25] revealed that pretrained embeddings can improve model performance in handling OOV. However, testing using contextual embeddings has not yet been conducted. We also plan to focus on overcoming the inconsistencies label in POS Tagging corpus. The similarity of annotator’s perception and consistency in word labeling is essential for POS tagging. Based on our previous research [26], we can conclude that corpus correction improves model performance. In addition, this study still uses data from one type of language (Indonesian). Further research can be developed for other agglutinative languages that are still in the same family, Austronesian such as Tagalog, Malay, and Javanese, considering that these languages have the same pattern.

The experimental results demonstrate that using the proposed feature fusion resulted in higher classification performance compared to the use of no features and single features. The proposed model, which uses a combination of orthographic, morphological, and character features, achieved the highest F1 score for both classification on all words and classification for only OOV words, with a macro-averaged F1 score of 85.79 % and 46.44 %, respectively. The scores were higher than those of the baseline model, which achieved a macro-averaged F1 score of 81.08 % and 37.65 % for classification on all words and classification for only OOV words, respectively. The use of softmax and CRF, did not yield significant differences in model performance.

## Ethics statements

This work did not involve human subjects, animal experiments data, and data collected from social media platforms.

## CRediT author statement

**Muhammad Alfian**: software, writing— original draft preparation; **Umi Laili Yuhana**: Conceptualization, methodology, Supervision, writing— original draft preparation; **Daniel Siahaan**: methodology, Supervision, writing—review & editing; **Harum Munazharoh**: data curation, Supervision, validation, writing—review & editing; **Eric Pardede**: Supervision, writing—review & editing

## Declaration of competing interest

The authors declare that they have no known competing financial interests or personal relationships that could have appeared to influence the work reported in this paper.

## Data Availability

I have share the link to my code at the Attach file step

## Appendix Supplementary materials

Image, application 1
